# Lerend monitoren en evalueren binnen gemeentelijke gezondheidsprojecten: inzichten op basis van elf projecten

**DOI:** 10.1007/s12508-025-00454-4

**Published:** 2025-02-14

**Authors:** Maud J. J. ter Bogt, Sid Penders, Gerdine A. J. Fransen, Jessica S. Gubbels

**Affiliations:** 1 Afdeling Eerstelijnsgeneeskunde, Radboudumc, Nijmegen, Nederland; 2 Academische werkplaats AMPHI Integraal gezondheidsbeleid, Nijmegen, Nederland; 3 Afdeling Gezondheidsbevordering, Research Institute of Nutrition and Translational Research in Metabolism (NUTRIM), Universiteit Maastricht, Maastricht, Nederland

**Keywords:** lerend monitoren en evalueren, gezondheidsbevordering, interprofessionele samenwerking, gemeenten, Learning-based monitoring and evaluation, Health promotion, Interprofessional collaboration, Municipalities

## Abstract

Veel Nederlandse gemeenten implementeren een of meer gezondheidsaanpakken, bijvoorbeeld rond roken, bewegen of gezond gewicht. Lerend Monitoren en Evalueren (LME) is hier vaak – in meer of mindere mate – onderdeel van. Dit artikel beschrijft hoe elf projecten binnen het ZonMw-programma Gemeenten Samen Gezond LME toepassen, en wat hierbij de ervaren kernelementen zijn: effectief diverse stakeholders erbij betrekken, cyclisch tot acties komen en bijsturen, continu monitoren en informatie verzamelen, LME (resultaten) borgen en continueren na de projectperiode, en LME (resultaten) overdragen naar andere contexten. We adviseren stakeholders LME toe te passen op een manier die past bij hun lokale context, doel en de betreffende onderzoeks-/praktijkvraag.

**Digitaal aanvullende content:**

De online versie van dit artikel (10.1007/s12508-025-00454-4) bevat aanvullend materiaal, toegankelijk voor daartoe geautoriseerde gebruikers.

## Abstract (en)

Numerous Dutch municipalities implement health promotion approaches focusing on themes such as smoking, exercising and healthy weight. Learning-based Monitoring and Evaluation (LME) is a crucial component that is often implemented as part of these approaches, to a larger or lesser degree. This article describes how eleven projects within the program Gemeenten Samen Gezond (‘Municipalities Healthy Together’) implement LME, and what perceived core elements of LME are: involving a diverse group of stakeholders, a process of cyclical action and adjustment, collecting information and monitoring, sustainability and continuation of LME and the program, and transferability to other settings. We recommend that stakeholders apply LME in a way that matches with their local context, purpose and research/practice question at hand.

## Inleiding

Het Gezond en Actief Leven Akkoord (GALA) onderstreept het belang dat rijksoverheid, gemeenten en zorgverzekeraars samen meer inzetten op het gebied van preventie en gezondheid om te werken aan gezondheidsproblemen [1]. In Nederland besteden veel gemeenten al aandacht aan preventie met (keten)aanpakken en projecten [1]. Deze projecten zijn vaak onderdeel van een regionale gezondheidsaanpak en worden uitgevoerd in een complexe omgeving, waarin verschillende factoren met elkaar en de aanpak interacteren.

Door deze complexiteit en interactie moeten gezondheidsaanpakken continu aangepast worden. Lerend monitoren en evalueren (LME) is dan ook essentieel om tussentijds te kunnen bijsturen, en zo de kans op effectiviteit te vergroten. LME omvat het continu ophalen en duiden van informatie over gezondheidsprojecten- en aanpakken om deze bij te sturen [2–4]. LME-betrokkenen ervaren echter diverse barrières, zoals beperkte tijd, beperkte kennis over LME of een mismatch tussen LME en de praktijk. Daarom is meer inzicht nodig in de manier waarop LME in gezondheidsaanpakken effectief kan worden toegepast [5].

In 2021 zijn twaalf projecten gestart binnen het ZonMw-programma Gemeenten Samen Gezond [6]. Deze richten zich op het versterken van een of meer bestaande gezondheidsaanpakken in hun regio en leveren diverse ervaringen op met laagdrempelig en continu LME in verschillende contexten. Dit artikel beschrijft hoe LME binnen deze ZonMw-projecten wordt toegepast en wat hierbij de ervaren kernelementen van LME zijn.

## Consultatie

Betrokkenen van elf van de twaalf Gemeenten Samen Gezond-projecten (91,7 % respons, *n* = 1 non response wegens personeelswisselingen) bundelden hun LME-ervaringen. Deze projectleden vertegenwoordigden de gemeentelijke praktijk vanuit kennisinstellingen, GGD’en en gemeenten. METC Oost-Nederland gaf een niet-WMO verklaring (aanvraagnummer 2023-16675). Alle deelnemers ontvingen een informatiebrief en ondertekenden een toestemmingsformulier. De dataverzameling bestond uit drie onderdelen die samen hebben geleid tot de beschreven kernelementen:

- Van elf projecten vulde een projectlid (diegene die door het betreffende project werd gezien als het meest betrokken bij LME) een korte vragenlijst in over hoe LME er in het project uitzag, wat de ervaren best practices en barrières waren, en welke leervragen hij of zij tijdens de bijeenkomst wilde uitdiepen (zie bijlage 1 in de digitaal aanvullende content).
- In december 2023 vond een Lerend monitoren en evalueren ZonMw-inspiratiesessie plaats. De achttien deelnemers uit de elf projecten en vertegenwoordiging vanuit ZonMw namen eraan deel. De leervragen uit de afgenomen vragenlijst werden aangevuld en vervolgens plenair geclusterd, overeenkomend met de hieronder beschreven kernelementen [7]. Vervolgens werden de clusters en onderliggende leervragen in subgroepen van deelnemers uitgediept en werden de verworven inzichten achteraf plenair teruggekoppeld.
- Na de inspiratiesessie ontvingen de aanwezigen een evaluatievragenlijst over de opgedane inzichten rond lerend monitoren voor hun project; ook werd gevraagd waar ze tegenaan blijven lopen (zie bijlage 2 in de digitaal aanvullende content). Deze vragenlijst werd door zeven deelnemers (van zeven projecten) ingevuld.

De kernelementen kwamen voort uit de consensus over de clusters van de inspiratiesessie en zijn door twee onderzoekers (MB, SP) waar nodig op basis van de vragenlijstendata aangevuld en opgesplitst. Ten slotte werd literatuur gezocht om de kernelementen te onderbouwen.

## Lerend monitoren en evalueren

Volgens de betrokkenen gaat LME over samen kennis opdoen tijdens de uitvoering van het project, om vervolgens het geleerde toe te passen door bij te sturen in de praktijk. Dit continue proces wordt samen met diverse professionals op het gebied van onderzoek, praktijk en beleid vormgegeven, waarbij idealiter ook inwoners betrokken zijn. LME wordt binnen de projecten op verschillende manieren vormgegeven. Het monitoren bestaat uit diverse methoden, waaronder formele methoden, zoals beleidsanalyse en actiebegeleidend participatief onderzoek, en informele methoden, zoals ‘koffiezetapparaatgesprekken’. Het delen van de LME-inzichten wordt in de praktijk veelal in de vorm van leernetwerken gedaan. In een leernetwerk komen diverse stakeholders die betrokken zijn bij (een onderdeel van) een gezondheidsaanpak geregeld samen om van elkaar te leren en de gezondheidsaanpak te versterken [4]. Er bestaan verschillende synoniemen van leernetwerken [2], zoals *communities of practice*, leerkringen, werkgroepen of kennistafels.

Aan de hand van de ervaringen van de deelnemende projecten werden vijf ervaren kernelementen van LME geïdentificeerd, die hieronder zijn toegelicht.

### Effectief diverse stakeholders erbij betrekken

Binnen een gemeentelijke gezondheidsaanpak zijn diverse stakeholders betrokken, denk aan beleidsstakeholders, inwoners of zorgprofessionals. Deze stakeholders zijn in meer of mindere mate ook betrokken bij LME [3, 8]. Dit kan ingewikkeld zijn, omdat diverse stakeholders verschillende leer- of samenwerkingsstijlen hebben (bijvoorbeeld vanuit de praktijk of vanuit een projectplan). Daarnaast kunnen multidisciplinaire stakeholders verschillende doelen hebben, waardoor ze soms ook verschillende LME-doelen hebben, bijvoorbeeld met betrekking tot andere onderwerpen (denk aan mentale gezondheid versus overgewicht). Ook zijn LME-doelen vaak aanvullend aan de doelen die in de gemeentelijke opdrachten van deze stakeholders genoemd worden, waardoor LME-doelen niet altijd regulier worden meegenomen in de werkzaamheden van de stakeholders. Dit kan nadelig zijn voor de betrokkenheid en het draagvlak van deze stakeholders bij LME en de gezondheidsaanpak.

Drie dingen kunnen helpen om, ondanks deze verschillen, diverse stakeholders erbij te betrekken. Ten eerste is het belangrijk om vanaf het begin verwachtingsmanagement in te zetten, concrete en behapbare (sub)doelen te stellen, en afspraken te maken [3]. De betrokkenheid wordt versterkt als dit periodiek wordt herhaald om, waar nodig, eerdere doelen en afspraken bij te stellen, zodat deze bij alle stakeholders blijven aansluiten. Ten tweede is het essentieel om een veilige omgeving met open, transparante communicatie te creëren. Het gebruik van diverse, creatieve werkvormen kan hierbij helpen. Zo maakt een van de projecten gebruik van kleuren (rood, oranje en groen) om de acties te evalueren en bij te sturen, wat leidt tot interactieve discussies tussen stakeholders. Deze methode dwingt stakeholders om te reflecteren op het functioneren van het collectief en op zichzelf. Ten slotte is een persoonlijke benadering essentieel om betrokkenheid te creëren en te behouden [4]. LME wordt dus idealiter afgestemd op de wensen en mogelijkheden van alle individuele LME-stakeholders. Bewoners kunnen bijvoorbeeld op een andere manier input leveren binnen het project dan beleidsstakeholders. Zo creëert een project bij de opstartfase van LME een deelproject met praktische taken voor ‘doeners’. Projectleiders van Gemeenten Samen Gezond benadrukken bovendien dat het gaat om een afgestemde combinatie van methoden.

### Cyclisch tot actie blijven komen en bijsturen

LME bestaat uit regelmatig samenkomen, zodat de stakeholders continu hun werkzaamheden op elkaar kunnen afstemmen en waar nodig kunnen bijsturen. Deze bijeenkomsten vormen een periodiek vinger-aan-de-pols-moment. Diverse projecten gebruiken hierbij een vorm van de eerder ontwikkelde *observe-reflect-plan-act*-cyclus [9]. Dit betekent dat opgehaalde kennis (observe) tijdens een bijeenkomst wordt teruggekoppeld aan de betrokken stakeholders. Deze kennis kan bestaan uit onderzoeksresultaten en/of voorbeelden uit de praktijk. Vervolgens reflecteren LME-betrokkenen op deze kennis (reflect), waarna ze plannen maken (plan) om het project verder te versterken in lijn met de gestelde doelen (act). Om dit te realiseren zijn passende werkvormen en methoden belangrijk [3, 7]. Zo laat een van de projecten soms een gezondheidsprofessional die niet direct bij het project betrokken is met een frisse blik meedenken. Daarnaast biedt goede verslaglegging een stok achter de deur, zodat kennis niet verloren gaat en geformuleerde plannen gezamenlijk vastgelegd worden. Vervolgens voeren LME-betrokkenen de plannen tussen de bijeenkomsten door uit. Hierbij is het belangrijk dat de LME-betrokkenen draagvlak en verantwoordelijkheid voelen. Dit kan versterkt worden door actief naar de mening van LME-betrokkenen te vragen en hierbij aan te blijven sluiten of door elkaar aan te spreken als gemaakte afspraken niet worden nagekomen. Een deelnemer raadt aan om het argument ‘geen tijd’ niet te snel als excuus te accepteren, maar te vragen wat de LME-betrokkene gaat doen om dit op te lossen. Naast observaties, reflecties en acties over de gezondheidsaanpak, dient dit ook te gebeuren voor het procesmatige deel van het project of LME, zodat ook deze (sub)doelen, afspraken of rollen kunnen worden bijgestuurd. Denk aan samen reflecteren op het programma en de deelnemers van een leernetwerkbijeenkomst.

### Continu monitoren en informatie verzamelen

Uniek aan LME is dat het monitoren van voor de praktijk relevante indicatoren samen en continu gebeurt. Er kan op verschillende niveaus gemonitord worden [2]. Zo kan informatie worden verzameld over de gehele gezondheidsaanpak (zoals het sociale netwerk van de gezondheidsaanpak) en/of een specifiek onderdeel (zoals een evaluatie van één interventie). Daarnaast kan bij het monitoren worden gefocust op het effect van een aanpak en/of op het proces. Ten slotte kan monitoren in meer of mindere mate worden vormgegeven vanuit het wetenschappelijk perspectief (top-down) en/of het praktijkperspectief (bottom-up). In de praktijk worden deze niveaus bij LME veelal gecombineerd, bijvoorbeeld door vragen bij LME-stakeholders op te halen en onderzoeksmethoden aan te laten sluiten bij hun leefwereld en vragen, waarbij de onderzoeker zorg draagt voor het gebruik van wetenschappelijke inzichten (zoals bestaande monitoringstools) en adequate uitvoering van de monitoring. Monitoring samen met alle LME-stakeholders vormgeven helpt ook bij het vinden van de balans tussen enerzijds planmatig en gestructureerd, en anderzijds adaptief en flexibel werken binnen LME. De meeste projecten combineren verschillende onderzoeksmethoden, zowel kwalitatief als kwantitatief. Voorbeelden van methoden zijn interviews, participatief actiebegeleidend onderzoek en gezondheidsenquêtes [2, 3]. Zo neemt een van de projecten een wijkenquête af bij een steekproef van wijkbewoners en worden aanvullend wijkbewoners geïnterviewd en geobserveerd die activiteiten organiseren of aan activiteiten deelnemen. Een ander project werkt planmatig aan acties door opgehaalde informatie met de betrokkenen te delen, om vervolgens gezamenlijk nieuwe acties te formuleren en bij te sturen.

### LME (resultaten) borgen en continueren na de projectperiode

De complexiteit en continue veranderingen van gezondheidsaanpakken maken het noodzakelijk dat de LME werkwijzen en/of resultaten worden geborgd in de bestaande gezondheidsaanpakken, zodat LME ook na de initiële looptijd van een project blijft plaatsvinden. Drie dingen kunnen helpen bij deze borging. Ten eerste is het belangrijk dat de betrokkenheid en motivatie bij LME-stakeholders behouden blijven, zie hiervoor ‘1. Effectief diverse stakeholders erbij betrekken’. Ten tweede zorgen sommige projecten voor stapsgewijze LME-borging, bijvoorbeeld door fasegewijs tastbare producten aan stakeholders over te dragen, zodat die hiermee aan de slag kunnen [3]. Denk aan het opleveren van tussentijdse factsheets om de onderzoeksresultaten op korte termijn met de praktijk te delen en om stakeholders de opbrengst van het onderzoek te laten zien. Zo heeft een project een magazine door en voor projectbetrokkenen uitgebracht, waarmee de resultaten zichtbaar worden gemaakt en betrokkenen worden geënthousiasmeerd. Ten derde helpt het om een stevige LME-projectleider te hebben die het mandaat heeft om de gezondheidsaanpak en het LME te sturen en stakeholders kan wijzen op gemaakte afspraken [8]. Ook een LME-stuurgroep of een betrokken opdrachtgever helpt bij de borging en continuering van LME. Sommige projecten hebben bijeenkomsten over LME (resultaten) met bestuurders om bestuurlijk draagvlak te creëren, kaders te bewaken en bestuurlijke acties te creëren.

### LME (resultaten) overdragen naar andere contexten

De projectleden geven aan dat het belangrijk is dat de LME-werkwijzen en/of -resultaten worden overgebracht naar andere contexten, zoals een ander(e) gemeente, organisatie, thema of doelgroep. De ene context is echter de andere niet [10]. Contexten verschillen bijvoorbeeld wat betreft demografische kenmerken van de inwoners, de gemeentelijke beleidspunten of de manier waarop de context is georganiseerd. Daarom is het belangrijk om een stappenplan te ontwikkelen dat uitlegt hoe LME-werkwijzen en/of -resultaten in een andere context anders kunnen worden georganiseerd.

Het stappenplan voldoet idealiter aan drie kenmerken. Ten eerste: benoem de werkzame elementen van de LME-werkwijzen en/of -resultaten van de aanpak. Deze werkzame elementen vormen de vaste kern en zijn essentieel voor de effectiviteit wanneer ze in een andere context worden geïmplementeerd. Ten tweede: benoem de veranderbare elementen en de manier waarop deze passend zijn gemaakt voor de context. Hierdoor kunnen anderen deze processen overnemen of aanpassen aan een manier die past bij hun eigen context. Ten derde: zorg ervoor dat het stappenplan toegankelijk is, bijvoorbeeld door passend en eenvoudig taal- en beeldgebruik, en een adequate introductie van het stappenplan. Bestaande stappenplannen uit andere projecten kunnen hierbij inspiratie bieden.

## Conclusie: lerend karakter leidt tot concrete adequate acties

Het hoofddoel van LME is dat door het lerende karakter concrete, adequate acties in gang gezet worden. Hierbij zijn vijf kernelementen essentieel: effectief diverse stakeholders erbij betrekken, cyclisch tot acties komen en bijsturen, continu monitoren en informatie verzamelen, LME (resultaten) borgen en continueren na de projectperiode, en LME (resultaten) overdragen naar andere contexten (zie fig. 1). Elk project in het huidige onderzoek past LME in enige vorm toe, passend bij de betreffende context, het doel en de onderzoeks-/praktijkvraag.

**Figuur 1:**
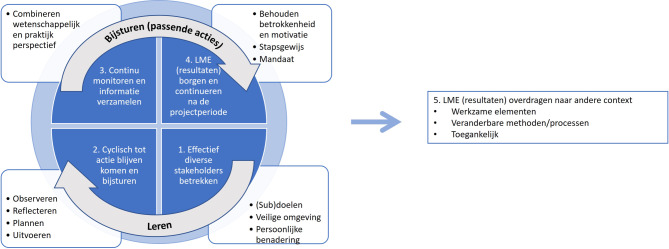
Kernelementen van lerend monitoren en evalueren in de praktijk

We adviseren gezondheidsbevorderaars om de vijf LME-kernelementen in een passende vorm te integreren in nieuwe én bestaande projecten en samenwerkingen, waarbij ervoor wordt gezorgd dat LME een zo groot mogelijke toegevoegde waarde heeft voor alle stakeholders binnen het project of de samenwerking. Hoewel we – wegens de diversiteit van geïncludeerde projecten – vermoeden dat de kernelementen representatief zijn voor diverse gezondheidsprojecten, adviseren we verder onderzoek naar de manier waarop gezondheidsprojecten LME (verder) kunnen worden toegepast.

## Digitaal aanvullende content

Bijlage 1: Korte vragenlijst
Bijlage 2: Evaluatie vragenlijst
