# Supplementary material for: Learning-based monitoring and evaluation within municipal health approaches: insights based on eleven projects
Source: TSG. 2025 Feb 14;103(1):15–9. [Article in Dutch] doi: 10.1007/s12508-025-00454-4 (PMC11914307; doi:10.1007/s12508-025-00454-4)
Supplement: Supplementary file 1 — Bijlage 1: Korte vragenlijst [file 12508_2025_454_MOESM1_ESM.docx]

**Bijlage 1: Korte vragenlijst**

1. Wat is de naam van jullie Gemeenten Samen Gezond project?
2. Wat is lerend monitoren voor jou?
3. Wat is het doel van jouw Gemeenten Samen Gezond project?
4. Hoe ziet lerend monitoren in jouw project er nu uit? Wat monitor je? Hoe monitor je het?
5. Hoe draagt lerend monitoren bij aan de uitvoer van je project?
6. Waar loop je tegen aan op het gebied van lerend monitoren?
7. Wat van wat je doet binnen lerend monitoren zou je andere projecten ook aanbevelen?
8. Wat wil je leren tijdens de bijeenkomst over lerend monitoren?
