# Supplementary material for: Learning-based monitoring and evaluation within municipal health approaches: insights based on eleven projects
Source: TSG. 2025 Feb 14;103(1):15–9. [Article in Dutch] doi: 10.1007/s12508-025-00454-4 (PMC11914307; doi:10.1007/s12508-025-00454-4)
Supplement: Supplementary file 2 — Bijlage 2: Evaluatie vragenlijst [file 12508_2025_454_MOESM2_ESM.docx]

**Bijlage 2: Evaluatie vragenlijst**

1. Wat is de naam van jullie Gemeenten Samen Gezond project?
2. Wat neemt u mee van vandaag omtrent het thema lerend monitoren en evalueren?
3. Wat gaat u hier concreet mee doen binnen je eigen project op het gebied van lerend monitoren en evalueren?
4. Wat mist u nog om met lerend monitoren en evalueren aan de slag te gaan binnen uw eigen project?
